# Cardiac Diastolic Evaluation in Pregnant Women with Abnormal Glucose Tolerance: An Opportunity to Detect the Early and Subclinical Alterations and Prevent Cardiovascular Diseases

**DOI:** 10.1155/2013/486593

**Published:** 2013-09-19

**Authors:** B. Pintaudi, G. Di Vieste, F. Corrado, M. F. Creazzo, A. Fazio, A. Valenti, R. D'Anna, A. Di Benedetto

**Affiliations:** ^1^Department of Internal Medicine, University of Messina, Via Consolare Valeria 1, 98125 Messina, Italy; ^2^Department of Obstetrics and Gynecology, University of Messina, Via Consolare Valeria 1, 98125 Messina, Italy; ^3^Department of Pathology and Experimental Micropathology, University of Messina, Via Consolare Valeria 1, 98125 Messina, Italy

## Abstract

Objectives of this study were to assess diastolic function in pregnant women with abnormal glucose tolerance (AGT), compared with normal glucose tolerance (NGT) women, and to evaluate the insulin resistance status and its association with Doppler-echocardiographic indexes. Echocardiograms of 108 consecutive Caucasian women with singleton pregnancies were performed. Insulin resistance status was estimated by the homeostasis model assessment of insulin resistance (HOMA-IR) and the quantitative insulin sensitivity check index (QUICKI). All the studied women showed normal diastolic patterns. Patients with AGT (50.9%), as compared with NGT women, had higher HOMA-IR (1.70 ± 1.30 versus 1.01 ± 0.81, *P* = 0.003), lower QUICKI (0.36 ± 0.005 versus 0.40 ± 0.06, *P* = 0.004), higher lateral mitral annulus late diastolic velocity (13.6 ± 4.9 versus 11.9 ± 4.9, *P* = 0.03), and higher A-wave velocity, the wave responsible for the active atrial contraction component (75.2 ± 14.2 versus 67.7 ± 16.2, *P* = 0.01). At multivariate regression analysis HOMA-IR was the only parameter associated with A-wave velocity. In conclusion, women with AGT had an increased subclinical diastolic active participation, which is associated with higher levels of insulin resistance. For the increased risk of deterioration of cardiac diastolic function, earlier and more seriously than normal pregnancy, AGT women may have a careful followup to detect the early signs of cardiac alteration and to prevent cardiovascular diseases.

## 1. Introduction

Abnormal glucose tolerance (AGT) in pregnancy represents a condition in which an alteration of glucose metabolism in pregnancy is detectable. It includes gestational diabetes mellitus (GDM) cases but also women with one abnormal value (OAV) at the oral glucose tolerance test (OGTT). GDM is the most frequent complication in pregnancy and represents an important risk factor for the onset of type 2 diabetes (T2DM) [1], hypertension, and cardiovascular disease (CVD) in the following years after the pregnancy [2]. Pregnant women with OAV, as women with GDM, are exposed to an increased risk for adverse perinatal outcomes [3, 4]. According to the new diagnostic criteria for GDM [5], even the presence of one altered blood glucose value at the OGTT allows a diagnosis of GDM. 

Recently, it has been assumed that cardiovascular impairment could start during pregnancy. In fact, frequently several markers of CVD risk, such as inflammatory factors, dyslipidemia, and hypertension, are present from the first moment of pregnancy [6, 7]. For these reasons it is extremely important to detect AGT cases in order to prevent all the complications related to this condition. Many studies in which cardiac function in normal pregnancy was evaluated have been conducted, but only few data are reported about cardiac function, in particular diastolic function, in pregnancies complicated by AGT. More specifically, left ventricular (LV) diastolic function of pregnant women was assessed by measurement of transmitral inflow velocity by pulsed wave Doppler echocardiography [8–11]. This method measures diastolic flow velocity, as index of LV diastolic capacity, but is strongly influenced by ventricular loading conditions. Furthermore, pregnancy is characterized by haemodynamic adaptations for which transmitral flow changes are not a valid reflection of diastolic function [12]. For this reason, tissue Doppler imaging (TDI), an established preload independent echocardiographic technique, is a more accurate method for the evaluation of diastolic function during pregnancy [13]. Given these premises, the principal aim of this observational study was to assess the diastolic function in pregnant women with AGT compared to a control group of pregnant women with normal glucose tolerance (NGT). In addition, the study aimed to evaluate the between group differences in the insulin resistance status and the association between metabolic parameters and Doppler-echocardiographic indexes.

## 2. Materials and Methods

### 2.1. Study Population

From March 2008 to January 2009, data on 108 consecutive Caucasian women with singleton pregnancies at the 29 ± 3 week gestation, with an abnormal result on a 50 g glucose challenge screening test (GCT), were evaluated. All participants performed a 3 h 100 g diagnostic OGTT for the determination of glucose tolerance status. Exclusion criteria were the following: the presence of any cardiac signs or symptoms, the history of cardiovascular disease, the diagnosis of diabetes mellitus before pregnancy and any treatment with corticosteroid agents, thyroid hormones, and medications known to modify cardiac structure or function. In particular, women with previous diastolic dysfunction and women with a previous pathological echocardiographic examination were carefully excluded from the study. All patients gave written informed consent to participate in the study.

### 2.2. Baseline Evaluation and Laboratory Measurements

All OGTTs were performed in the morning after an overnight fast. Glucose and insulin levels at fasting and at 60, 120, and 180 minutes after the glucose load were measured. Glucose tolerance during pregnancy was defined according to Coustan and Carpenter criteria [14]. Briefly, these criteria suggested an evaluation for GDM to be performed between 24 and 28 weeks' gestation in those women not known to have carbohydrate intolerance before the 24th week of gestation. This evaluation was done in a two-step procedure, consisting in a 50 g oral glucose challenge test (GCT), followed by a diagnostic 3 h 100 g OGTT if results of the GCT exceed a predetermined plasma glucose concentration (≥140 mg/dL one hour after ingestion of the glucose load). The cut-off glucose values for the diagnostic OGTT were: fasting ≥95 mg/dL, 1 h postload ≥180 mg/dL, 2 h postload ≥155 mg/dL, and 3 h postload ≥140 mg/dL. Two or more of the venous plasma concentrations must be met or exceeded for a positive diagnosis. Women with only one abnormal value for the 100 g 3 h OGTT (OAV) are considered to have the same risk as women with normal OGTT results [4].

The insulin sensitivity index homeostasis model assessment of insulin resistance (HOMA-IR) was calculated according to Matthews' formula [15]. The quantitative insulin sensitivity check index (QUICKI) was also calculated to estimate the insulin resistance [16]. Furthermore, clinical and obstetrical data were collected. Prepregnancy BMI and BMI at first prenatal visit were calculated. Blood pressure was measured after a 10-minute rest. All the patients were invited to return to our department at a future day for ECG and echocardiographic examinations.

### 2.3. Echocardiography

Echocardiograms were recorded with a commercially available ultrasound system (General Electrics VIVID 3 PRO) equipped with a 3.5 MHz phased-array transducer at 29.7 ± 2.6 week of pregnancy in group A and at 28.9 ± 4.0 week in group B. Subjects were examined in the left lateral decubitus position using standard parasternal, short-axis, and apical views. All recordings and measurements were obtained by the same operator who was blinded to the clinical data of the pregnant women. To define the reproducibility of echocardiography, parameters were examined offline from the digitally retrieved cycles in a random order by an independent operator. Two operators independently performed all measurements. Exams, averaging over three representative cardiac cycles, were performed after a 15 minute rest, in the postprandial state to avoid the influence of circadian rhythm on LV diastolic function [17]. LV ejection fraction was calculated by the Teicholz formula [18] in the absence of significant mitralic regurgitation and regional wall motion abnormalities. LV diastolic function was evaluated through three parameters: (a) by recording transmitralic flow in the zone of maximum mitralic flow by placing the sample gate of pulsed doppler at the tips of the mitral leaflets in their fully-open position in diastole, thus recording the wave expression of rapid LV filling (*E*) and the wave subordinate to the atrial contraction which is an expression of late LV filling (*A*) [19]; (b) by analysing *E* and *A* peaks and the *E*/*A* ratio; (c) by evaluating early (*E*′) and late (*A*′) diastolic velocity waves recorded using TDI module by placing the sample gate at the lateral part of mitral annulus, as a result, calculating the *E*′/*A*′ average. Finally *E*/*E*′ ratio, a measure of LV end-diastolic pressure, was calculated. The following parameters were also evaluated: left atrium anteroposterior diameter, left atrium area, interventricular thickness, LV diastolic diameter, LV posterior wall thickness, and pulmonary artery systolic pressure. Cardiac diastolic function was evaluated according to the current diagnostic criteria [20, 21].

### 2.4. Statistical Analyses

In descriptive analyses, continuous variables are summarized as mean and standard deviation (normal distribution) or median (nonnormal distribution and ordinal variables). Categorical variables are expressed as percentages. Differences among the groups were analyzed by analysis of variance (ANOVA) or chi-square tests. Pearson's correlation coefficient was employed to test correlations between HOMA-IR, QUICKI, and significantly different cardiovascular markers. Multivariate regression analyses were performed to analyze the effect of insulin resistance on the diastolic function indices. The following baseline covariates were tested: BMI at the echocardiogram, age, gestational age at echocardiograms, and HOMA-IR. *P* values < 0.05 were considered statistically significant. Statistical analysis was performed with SPSS statistical package version 17 (SPSS, Chicago, IL, USA).

## 3. Results

### 3.1. Clinical Data

Clinical characteristics of study participants are shown in Table 1. Pregnant women with AGT, when compared with women with NGT, were older, had higher fasting glucose, higher fasting insulin levels, higher levels of HOMA-IR, lower QUICKI, and higher levels of diastolic blood pressure, and were more frequently at the first pregnancy. There was no significant difference between groups in prepregnancy BMI, BMI at the echocardiograms, systolic blood pressure, and percentages of women with familiarity for T2DM.

### 3.2. Echocardiographic Measurements (M-Mode and 2-Dimensional Mode)

Table 2 shows the M-mode and 2-dimensional mode measurements for LV and atrial cavity. There were no differences between groups in LV posterior wall thickness, LV end-diastolic diameter, LV ejection fraction, and left atrial diameter and area. Interventricular septum during diastole was significantly larger in group A compared to group B.

### 3.3. Doppler and Tissue Doppler Parameters

There were no statistical significant differences between groups in rapid filling wave (*E* wave), PAPs, lateral mitral annulus early diastolic velocity (*E*′), *E*/*E*′ ratio, and *E*/*A* ratio. Lateral mitral annulus late diastolic velocity (*A*′) was higher in group *A*, and *E*′/*A*′ ratio was lower in group B. The velocity of the wave which is responsible for the active atrial contraction component (*A* wave) was increased in group A, as reported in Table 3. When considering diastolic dysfunction grade, defined according *E*/*A* ratio value, no women showed any grade of diastolic dysfunction. The correlation analyses show a positive correlation between both systolic and diastolic pressure, transmitral *A* velocity, and HOMA-IR when considering the overall sample. We can see similar results when considering only AGT women, but not NGT women. Similar correlations but in a negative sense are evident as attended with QUICKI (Table 4). Multivariate regression analysis, adjusted for BMI at the echocardiogram, age, gestational age at echocardiograms, and HOMA-IR, and where the *A* wave was the dependent variable, showed that HOMA-IR was the only parameter associated with *A* wave velocity (*β* = −0.334, *P* = 0.039).

## 4. Discussion

The first result of our study was the evidence that all the examined women had a normal pattern of diastolic function. Both pregnant women with AGT and women with NGT showed in fact no detectable echocardiographic parameters indicative of diastolic dysfunction. The only between group echo differences were related to *A* and *A*′ velocities and *E*′/*A*′ ratio, and the group of women with AGT shows higher velocities of these waves, which are involved in active atrial contraction. To our knowledge there is only one previous study in which diastolic function in patients with AGT was analyzed [22]. In particular, Freire and colleagues showed a different LV diastolic filling profile when examining diastolic function in 13 young patients with GDM, recognizing some cases of diastolic dysfunction. Compared to their case study we analyzed a greater number of women who were furthermore at an earlier week of gestation. We did not find any case of diastolic dysfunction and this could be due to the earlier gestational time of our examination. We found diastolic patterns in a normal range but the highlighted subclinical differences between group could represent initial diastolic abnormalities that are present since an early phase of the pregnancy. 

We focused our study on cardiac diastolic function because its alteration is the first recognizable in case of diabetes-linked cardiopathy. The information provided by using transmitral annular tissue doppler data is useful in the assessment of diastolic dysfunction diagnosis. Pregnancy causes physiological hemodynamic adaptations since the first trimester [23] because total vascular resistance decreases, blood volume overloads, and heart rate and blood pressure can be decreased. All these changes contribute to influence mitral inflow indices and other Doppler diastolic parameters [24]. A better evaluation of LV diastolic function requires direct LV pressure and volume measurements by cardiac catheterization [25], which is not simple and ethically correct for clinical studies in healthy individuals. Now it is possible to evaluate diastolic function noninvasively using echocardiography with Doppler measurements of transmitral blood flow and more recently developed myocardial tissue doppler imaging (TDI) measurements [26, 27]. TDI evaluation has the big advantage of being less dependent on preload. It is demonstrated that, despite an apparent increase in function in early normal pregnancy, cardiac diastolic function, in the basal resting state, appeared to deteriorate by term [28, 29]. 

Many authors reported increased transmitral *A* velocity and *E*/*A* ratio during normal pregnancy [8–11, 29, 30]. We found a further increase of the values of the same parameters in AGT women, as compared with NGT women.

The other aim of our study was to assess insulin resistance status and its link with Doppler-echocardiographic indexes. Women with AGT had higher levels of insulin resistance, as measured by HOMA-IR, and lower level of insulin sensibility, as measured by QUICKY, than NGT women. A strong correlation between insulin resistance and heart alterations has been noted [31–33]. More epidemiological lines evidence of demonstrated that insulin resistance could predict the subsequent development of heart failure, determining cardiomyocyte hypertrophy, independent of all established risk factors, including diabetes mellitus itself [31]. The state of insulin resistance is characterized by elevated circulating free fatty acid levels that are stored as intramyocardial triglycerides and can negatively influence heart's oxidative capacity [34]. In our study we found a significant between group difference in interventricular septum thickness during diastole. This could be explained because, despite an increased thickness in normal pregnancy, in the group of women with AGT there was a higher insulin resistance status that have determined cardiomyocyte hypertrophy. Higher insulin resistance could reasonably set these women at higher risk of developing cardiac disease in the future. At multivariate analysis insulin resistance was the only parameter associated with A wave velocity, and this could represent the functional aspect of an increased insulin resistant role in promoting impaired subclinical diastolic cardiac function.

## 5. Conclusions

We found a higher subclinical diastolic active participation in AGT women as compared with NGT women. The significant differences in *A* wave, *A*′, and *E*′/*A*′ ratio could be explained by the potential effect of insulin resistance in deteriorating cardiac diastolic function of AGT women earlier and more seriously than normal pregnancy. For this reason and for the demonstrated future cardiovascular risk, women with GDM or OAV may have a careful followup to detect the early sign of cardiac dysfunction and to prevent heart failure. Limits of our study are the relatively low number of women examined, although in the only existing work a smaller number of women, compared to our, have been examined, and the lack of a cardiology followup after pregnancy can estimate the diastolic cardiac performance in these women with higher cardiovascular risk. 

## Figures and Tables

**Table 1:** Clinical characteristics of pregnant women with abnormal glucose tolerance (AGT) in pregnancy and pregnant women with normal glucose tolerance (NGT).

	Abnormal glucose tolerance woman	Normal glucose tolerance woman	*P* value
*N* (%)	55 (50.9)	53 (49.1)	ns
Age (yr)	34.6 ± 4.9*	31.6 ± 6.2	0.006
Prepregnancy BMI (Kg/m²)	24.7 ± 4.1	24.9 ± 5.9	ns
BMI at the echocardiograms (Kg/m²)	28.1 ± 4.2	27.1 ± 5.5	ns
Familiarity for type 2 diabetes, *n* (% )	28 (50.9)	27 (49.1)	ns
Previous pregnancies, *n* (% )	76 (63.1)*	59 (36.9)	0.003
Smoke during pregnancy, *n* (% )	5 (9.4)	5 (9.0)	ns
Gestational age at echocardiograms (weeks)	29.7 ± 2.6	28.9 ± 4.0	ns
Heart rate (beats/min)	87.0 ± 10.9	83.9 ± 10.7	ns
Systolic blood pressure (mmHg)	108.7 ± 10.1	105.3 ± 11.6	ns
Diastolic blood pressure (mmHg)	68.9 ± 8.91*	64.8 ± 8.9	0.02
Fasting glucose (mg/dL)	83.0 ± 15.4*	70.8 ± 10.2	0.02
Fasting insulin (*μ*mol/L)	8.8 ± 5.6*	6.3 ± 4.6	0.001
HOMA-IR	1.70 ± 1.30*	1.01 ± 0.81	0.003
QUICKI	0.36 ± 0.005*	0.40 ± 0.06	0.004
Women with OAV, *n* (%)	23 (21.3)	0 (0)	0.001

Data are expressed as means ± SD or percentages. *P values < 0.05 were considered statistically significant.

**Table 2:** Echocardiographic M-mode and 2-dimensional mode measurements.

	Abnormal glucose tolerance woman	Normal glucose tolerance woman	*P* value
*N* (%)	55 (50.9)	53 (49.1)	ns
Interventricular septum during diastole, IVSd (mm)	10.0 ± 1.1*	9.4 ± 1.3	0.009
Left ventricular end-diastolic diameter, LVEDD (mm)	46.2 ± 3.4	45.6 ± 3.1	ns
Left ventricular posterior wall thickness, LVPWd (mm)	9.9 ± 1.1	9.5 ± 1.2	ns
Ejection fraction, EF (%)	67.6 ± 5.8	68.0 ± 4.9	ns
Left atrial area (cm^2^)	15.4 ± 1.8	14.9 ± 1.8	ns
Left atrial diameter (mm)	36.2 ± 3.0	35.8 ± 2.9	ns

Data are expressed as means ± SD or percentages. *P values < 0.05 were considered statistically significant.

**Table 3:** Doppler and tissue Doppler parameters.

	Abnormal glucose tolerance woman	Normal glucose tolerance woman	*P* value
*N* (%)	55 (50.9)	53 (49.1)	Ns
Pulmonary artery pressure, PAP (mmHg)	21.3 ± 6.3	20.3 ± 6.7	Ns
Transmitral *E* velocity (cm/s)	81.0 ± 22.0	77.2 ± 20.0	Ns
Transmitral *A* velocity (cm/s)	75.2 ± 14.2*	67.7 ± 16.2	0.01
*E*/*A* ratio	1.1 ± 0.3	1.2 ± 0.3	Ns
Lateral *E*′ velocity (cm/s)	16.3 ± 3.2	17.3 ± 5.9	Ns
Lateral *A*′ velocity (cm/s)	13.6 ± 4.9*	11.9 ± 4.9	0.03
*E*/*E*′ ratio	5.0 ± 1.3	4.8 ± 1.5	Ns
*E*′/*A*′ ratio	1.3 ± 0.5*	1.7 ± 1.0	0.01

Data are expressed as means ± SD or percentages. *P values < 0.05 were considered statistically significant.

**Table 4:** Correlation analyses between HOMA-IR, QUICKI, and significantly different cardiovascular markers.

	OVERALL woman		AGT woman		NGT woman	
	*R*	*P*	*R*	*P*	*R*	*P*
HOMA-IR						
Systolic blood pressure	0.304	0.003	0.325	0.03	0.220	0.13
Diastolic blood pressure	0.439	<0.0001	0.445	0.002	0.338	0.02
Interventricular septum during diastole	0.199	0.05	0.075	0.62	0.214	0.15
Transmitral *A* velocity	0.309	0.002	0.291	0.05	0.128	0.39
Lateral *A*′ velocity	−0.022	0.83	−0.037	0.81	−0.201	0.18
*E*′/*A*′ ratio	−0.015	0.89	−0.011	0.94	0.141	0.34
QUICKI						
Systolic blood pressure	−0.183	0.08	−0.218	0.15	−0.093	0.53
Diastolic blood pressure	−0.354	<0.0001	−0.426	0.003	−0.218	0.14
Interventricular septum during diastole	−0.158	0.13	−0.138	0.36	−0.052	0.73
Transmitral *A* velocity	−0.367	<0.0001	−0.348	0.02	−0.256	0.08
Lateral *A*′ velocity	−0.029	0.78	−0.048	0.75	0.108	0.47
*E*′/*A*′ ratio	0.027	0.80	0.011	0.95	−0.075	0.62
